# Multimorbidity worsened anxiety and depression symptoms during the COVID-19 pandemic in Brazil

**DOI:** 10.1016/j.jad.2022.07.005

**Published:** 2022-10-01

**Authors:** Luis Fernando Silva Castro-de-Araujo, Elisângela da Silva Rodrigues, Daiane Borges Machado, Claudio Maierovitch Pessanha Henriques, Mariana Pastorello Verotti, Alessandra Queiroga Gonçalves, Talita Duarte-Salles, Richard A. Kanaan, Mauricio Lima Barreto, Glyn Lewis, Jakeline Ribeiro Barbosa

**Affiliations:** a Center of Data and Knowledge Integration for Health (CIDACS), Fiocruz, Salvador, Brazil; b Federal University of Ceará, Campus Jardins de Anita, Itapajé, Ceará, Brazil; c Harvard Medical School, Department Global Health and Social Medicine, United States of America; d Center for Epidemiology and Health Surveillance, Oswaldo Cruz Foundation, Brasília, Brazil; e Unitat de Suport a la Recerca Terres de l'Ebre, Fundació Institut Universitari per a la recerca a l'Atenció Primària de Salut Jordi Gol i Gurina (IDIAPJGol), Tortosa, Catalunya, Spain; f Unitat Docent de Medicina de Família i Comunitària Tortosa-Terres de L'Ebre, Institut Català de la Salut, Tortosa, Catalunya, Spain; g Fundació Institut Universitari per a la recerca a l'Atenció Primària de Salut Jordi Gol i Gurina (IDIAPJGol), Barcelona, Spain; h Dept of Psychiatry, The University of Melbourne, Austin Health, Victoria, Australia; i Division of Psychiatry, UCL, London, United Kingdom of Great Britain and Northern Ireland

**Keywords:** Covid-19, Pandemic, Chronic diseases, Mental disorders, Epidemiology, Physical exercises, Depression, Anxiety

## Abstract

Multimorbidity is a global health issue impacting the quality of life of all ages. Multimorbidity with a mental disorder is little studied and is likely to have been affected by the COVID-19 pandemic. We used a survey of 14,007 respondents living in Brazil to investigate whether people who already had at least one chronic medical condition had more depression and anxiety symptoms during social distancing in 2020. Generalized linear models and structural equation modelling were used to estimate the effects. A 19 % and 15 % increase in depressive symptoms were found in females and males, respectively, for each unit of increase in the observed value of reported chronic disease. Older subjects presented fewer symptoms of depression and anxiety. There was a 16 % increase in anxiety symptoms in females for each unit increase in the reported chronic disease variable and a 14 % increase in males. Younger subjects were more affected by anxiety symptoms in a dose-response fashion. High income was significantly related to fewer depressive and anxiety symptoms in both males and females. Physical activity was significantly associated with fewer anxiety and depression symptoms. Structural equation modelling confirmed these results and provided further insight into the hypothesised paths.

## Highlights

• To evaluate if the presence of chronic diseases rises the risk of reporting anxiety or depression symptoms in the pandemic
• It was found that this association existed and was significant for both anxiety and depressive symptoms
• High income was significantly related to fewer depressive and anxiety symptoms in both males and females

## 1 Introduction

Multimorbidity, the presence of two or more chronic health conditions, is a global health priority, affecting patients of all ages and significantly impacting the quality of life (The Academy of Medical Sciences, 2018). Mental disorders are among the most common chronic conditions, and commonly affect the course and severity of other comorbid conditions. The worldwide prevalence of chronic physical illness among people living with mental disorders, and the number of years of living with disability, are increasing (Filipčić et al., 2018; Firth et al., 2019).

A recent review noted how little research was being performed on mental health in multimorbidity (Firth et al., 2019). Information on multimorbidity that includes at least one mental disorder is currently fragmented. This hinders effective identification of at-risk groups and prompts clinical action towards limiting adverse consequences in multimorbid patients.

There is evidence of increased risk of mental disorders, such as depression, in subjects with chronic health conditions (Read et al., 2017; Rzewuska et al., 2017). Two meta-analysis have shown an increased risk of depression (Read et al., 2017) and psychosis (Rodrigues et al., 2021) among multimorbid individuals, although the latter reported wide range of prevalence estimates, possibly linked to the varied definitions of multimorbidity used by the studies included in the analysis. Conversely, people with mental disorders are often at increased risk of physical health conditions, such as metabolic syndrome in schizophrenia (Maj et al., 2021), in part due to treatment (Tandon et al., 2010). Associations between mental disorders and chronic conditions involving multiple organs or systems have been identified, such as type 2 diabetes, chronic pulmonary obstructive disease (Filipčić et al., 2018), and arthritis with depression (Teh et al., 2018). Subjects with mental disorders are also at higher mortality risk from COVID-19 (Wang et al., 2021). Furthermore, individuals with multimorbidity had higher risk of presenting anxiety symptoms (Azevedo et al., 2022).

The COVID-19 pandemic has led to distress, economic instability and isolation, and has made many people vulnerable to mental health problems and suicidal behaviour. The mental health consequences of these unprecedented circumstances are likely to endure and peak later than the pandemic itself (Gunnell et al., 2020). The mental health effects of social distancing are becoming better understood. It has been shown to increase anxious and depressive symptoms (Machado et al., 2020). For example, the prevalence of anxiety during the Chinese quarantine was 35.1 % (Huang and Zhao, 2020), with significantly higher levels in those younger than 35. The elderly are at higher risk of morbidity and death from SARS-CoV-2 infection (Castro-de-Araujo and Machado, 2020; Grassi et al., 2020).

Lockdowns were associated with both depressive and anxiety symptoms. A British prospective longitudinal study (Fancourt et al., 2021) confirmed what was found in surveys during the pandemic (Shah et al., 2021; Wu et al., 2021) that these symptoms were more severe during the start of the lockdowns and declined with time. Women, individuals with lower income, pre-existing mental health conditions, and individuals living alone had higher risk of presenting such symptoms (Fancourt et al., 2021).

To date, it is unknown whether subjects with pre-existing chronic conditions are at higher risk of reporting depressive or anxious symptoms during the pandemic. In this study, we aimed to investigate the association between the report of an existing chronic condition before the pandemic and psychiatric symptomatology during the pandemic using a large survey study during the COVID-19 pandemic in Brazil.

## 2 Methods

### 2.1 Study design and data collection

The data presented here is part of a larger mixed-methods international project studying the social impact of social distancing during the COVID-19 pandemic (Jacques-Aviñó et al., 2020). The data used for this study come from the first stage of this project, which comprised an online questionnaire administered from June, 20th to August, 30th in 2020. Quarantining was never enforced anywhere in Brazil. Regardless of the terminology regional governments used, there was no stay at home enforcement. In this work, we will refer to this phase as the social distancing phase.

In order to circulate the questionnaire, a virtual snowball (Costa, 2018) method was adopted, which consists of sending the access link of the electronic questionnaire via email to the virtual social network of the institution and researchers involved. This technique is a form of non-probabilistic sampling used in social surveys where the initial participants of a study indicate new participants who in turn indicate new participants and so on.

### 2.2 Study sample

All persons aged 18 years or over residing in Brazil during the COVID-19 pandemic, were eligible to participate in this study. Participation consisted of filling out an online form using a cell phone or computer with internet access.

### 2.3 Data collection

The online questionnaire, containing questions about socioeconomic, behavioural, confinement and health conditions, risk perception and emotional health in the context of confinement (Supplementary material), was prepared using the SurveyMonkey® platform, which consists of a digital platform for data collection, management and dissemination.

The recruitment of participants was carried out virtually by each of the participating centres in the countries involved in the research. In the case of Brazil, the SurveyMonkey® online survey software was used., The electronic questionnaire was disseminated through Oswaldo Cruz Foundation (Fiocruz) websites, postgraduate programs, Fiocruz state/regional units, public universities, and the researchers involved (Twitter, Instagram, WhatsApp and Facebook). Additionally, a report was published in print and broadcasted on television with local and regional (Central-West and Southeast) coverage in the country.

### 2.4 Variables

The independent variables used in the current analyses were: whether the respondent was aware of any previous chronic conditions (yes/no), respondent age, whether the respondent was quarantined/practising social distancing (No Social distancing/I am no longer confining/Social distancing), knowledge of any previous mental disorder (yes/no), whether the respondent had any mental health assistance prior to the pandemic (yes/no), the respondent's income (<2, 2–5, 5–10, >10 minimal wages), and change in the level of physical activity during the pandemic (no activity or decreased, the same, or increased during the pandemic). The dependent variables were scores for symptom severity.

Depressive symptomatology was assessed through the PHQ-9 scale (Spitzer et al., 1999) and anxious symptomatology via GAD-7 scale (Spitzer et al., 2006), both validated in Portuguese (Sousa et al., 2015; Torres et al., 2016).

### 2.5 Data analysis

Descriptive statistics were extracted from all variables. Continuous variables were analysed with the average of the descriptive measures, median, minimum, maximum and standard deviation (SD) and categorical variables were described with the total number of observations and their corresponding percentages stratified by sex. All analyses were performed using R version 3.6.3 (https://cran.r-project.org/).

The overall missingness of the variables used was around 11 % (Supplemental material), and its pattern was not completely at random according to Little's test (Little, 1988). Missing data were completed with simple imputation for the regressions and by pooling from 5 multiply imputed data frames using the R mice package with the classification and regression trees algorithm (van Buuren and Groothuis-Oudshoorn, 2011).

Two analytical strategies were used in order to investigate the relationship between reported previous chronic disease and psychiatric symptomatology during the pandemic. First, responses to PHQ-9 and GAD-7 were each included in a model to obtain a score in the depressive and anxiety dimensions. This was performed through a confirmatory factor analysis with a latent variable reflecting the variances from each of the responses in the scales. By using the R lavaan package (version 0.6–7) (Rosseel, 2012) we computed estimated values for the latent variables in the models, which can be obtained per subject. These scores were then outcomes in generalized linear modelling regressions with the reported presence or absence of previous chronic diseases as an independent variable and controlled by age strata, personal income, existing psychiatric disorder prior to the pandemic, whether the patient reported practising social distancing, and change in physical activities. We report the results of these models using Average Marginal Effect (AME), which are the mean change in probability in the dependent variable for each 1 unit increase in the explanatory variable.

### 2.6 Structural equation models

A second approach used was a structural equation model using the same variables that were included in the regressions, but now with the theoretical effect directions specified in paths.

Two models were specified with similar structures, each predicting a reflective latent variable representing either depressive or anxious symptoms. The reported presence of existing chronic disease was specified as an independent variable, the remaining variables from the regressions were also included. Both models were estimated using the robust version of weighted least squares (Fig. 3, Fig. 4). Standardized coefficients are interpreted as the amount of standard deviation change in the variable at the tip of the arrow, for each 1-SD change at the base of the arrow.

The variables included in the model were categorical and SEM is traditionally designed for continuous variables. In order to work with categories, one has to imply that the observed categorical variable is indicative of a latent (unobserved) continuous variable (commonly referred to as liability). The distribution of the liability is expected to have one or more thresholds, thus allowing calculation of standard deviation in relation to the independent variables (Beaujean, 2014). This is the case of the variable encoding reported chronic condition which was thresholded to allow estimation of coefficients (standard deviation change) in relation to the other indicators in the structural specification.

### 2.7 Ethics

The study was approved by the National Research Ethics Commission (Conep) on June 17, 2020, under registration CAAE 31010820.6.0000.8027. At the beginning of the electronic questionnaire, participants were required to read the details of the research in an informed consent term (IC, Supplementary material). This was electronically signed by selecting the option to accept the invitation. A copy was also made available electronically to the participant. The research follows the recommendations of the Declaration of Helsinki and Tokyo. The SurveyMonkey® Platform used for virtual data collection is secure, with data stored on a restricted access and unique key server. The study complies with the General Law for the Protection of Personal Data in Brazil - LGPDP (Law No. 13,709 of August 14, 2018).

## 3 Results

A total of 14,007 subjects answered the survey. Our data was unbalanced in regard to sex. 11,010 (78.6 %) of the participants were women and there were significant differences in the depressive and anxious symptom scores between sexes. Also, 723 respondents did not answer the question about biological sex. Female and male respondents differed significantly in PHQ-9 scores, the mean score was respectively 8.25 (SD 5.65) and 6.87 (SD 5.74). They also differed significantly in GAD7 scores, the mean for females was 7.93 (SD 4.85) and 6.57 (SD 5.07) for males (Table 1). There were also statistically significant differences in chronic conditions, age, social isolation status, mental disorders, income, and physical activity level between sexes.

**Table 1:** Main demographic characteristics of the sample. Group comparison was performed using chi-2 tests for the categorical variables and ANOVA for the continuous variables (anxiety, depression scores). Note that 723 respondents did not answer the question about biological sex, hence the difference between the total and each column.

n (%)	Total 14,007	Female 11,010	Male 2914	P
Reported chronic disease (%)				<0.001
No	6110 (43.6)	4720 (42.9)	1357 (46.6)	
Yes	5638 (40.3)	4567 (41.5)	1038 (35.6)	
NA	2259 (16.1)	1723 (15.6)	519 (17.8)	
Age (%)				<0.001
[18,33]	3738 (26.7)	2810 (25.5)	897 (30.8)	
[33,43]	3316 (23.7)	2685 (24.4)	620 (21.3)	
[43,56]	3568 (25.5)	2870 (26.1)	682 (23.4)	
[56,98]	3385 (24.2)	2645 (24.0)	715 (24.5)	
Color (%)				0.102
White	9447 (67.4)	7486 (68.0)	1917 (65.8)	
Black	896 (6.4)	697 (6.3)	191 (6.6)	
Others	3565 (25.5)	2747 (25.0)	788 (27.0)	
NA	99 (0.7)	80 (0.7)	18 (0.6)	
Region (%)				<0.001
Central-West	1543 (11.0)	1139 (10.3)	392 (13.5)	
North	389 (2.8)	289 (2.6)	100 (3.4)	
Northeast	1627 (11.6)	1208 (11.0)	416 (14.3)	
South	2277 (16.3)	1832 (16.6)	432 (14.8)	
Southeast	8118 (58.0)	6513 (59.2)	1561 (53.6)	
NA	53 (0.4)	29 (0.3)	13 (0.4)	
Confinement (%)				<0.001
No social distancing	1647 (11.8)	1173 (10.7)	462 (15.9)	
I am no longer confining	3366 (24.0)	2666 (24.2)	683 (23.4)	
Social distancing	8961 (64.0)	7143 (64.9)	1766 (60.6)	
NA	33 (0.2)	28 (0.3)	3 (0.1)	
Reported mental disorder (%)				<0.001
No	9843 (70.3)	7653 (69.5)	2137 (73.3)	
Yes	2121 (15.1)	1792 (16.3)	314 (10.8)	
NA	2043 (14.6)	1565 (14.2)	463 (15.9)	
Psy assistance (%)				<0.001
No	9350 (66.8)	7245 (65.8)	2056 (70.6)	
Remote psy assistance	1960 (14.0)	1678 (15.2)	270 (9.3)	
In-person psy assistance	590 (4.2)	471 (4.3)	114 (3.9)	
NA	2107 (15.0)	1616 (14.7)	474 (16.3)	
Income - minimal wage (%)				<0.001
No	2571 (18.4)	2055 (18.7)	497 (17.1)	
<2	3024 (21.6)	2397 (21.8)	607 (20.8)	
2–5	4035 (28.8)	3298 (30.0)	715 (24.5)	
5–10	2669 (19.1)	2104 (19.1)	550 (18.9)	
>10	1639 (11.7)	1106 (10.0)	528 (18.1)	
Physical activity (%)				<0.001
No	2632 (18.8)	2206 (20.0)	414 (14.2)	
Decreased	6139 (43.8)	4778 (43.4)	1324 (45.4)	
Same	1909 (13.6)	1434 (13.0)	463 (15.9)	
Increased	1475 (10.5)	1186 (10.8)	282 (9.7)	
NA	1852 (13.2)	1406 (12.8)	431 (14.8)	
PHQ9 (mean (SD))	7.97 (5.70)	8.25 (5.65)	6.87 (5.74)	<0.001
GAD7 (mean (SD))	7.65 (4.92)	7.93 (4.85)	6.57 (5.07)	<0.001

**Fig. 1:**
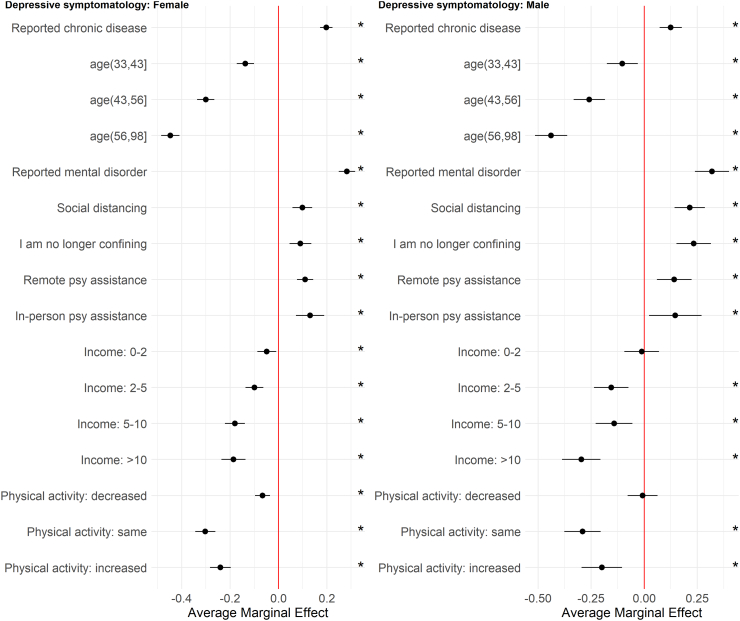
Results of glm models using Average Marginal Effect (AME). AME is the mean change in probability in the dependent variable for 1 unit increase in the explanatory variable. *, p < 0.05.

The report of a previous chronic condition was significantly associated with reporting of worse depressive symptomatology during social isolation in both males and females (Fig. 1). An AME of 0.19 was found between reported previous chronic disease and depressive symptoms, which means there was a 19 % increase in depressive symptoms in females for each unit change in the observed value (reported chronic disease) and 15 % increase in males. Age was protective in regard to depressive symptomatology, with older subjects having fewer symptoms. For example, for male subjects between 56 and 98 years of age there was a 46 % reduction in depressive symptomatology. Male and female subjects with an existing mental disorder were also at higher risk of presenting depression symptoms during confinement. Income was significantly related to fewer depressive symptoms during social distancing in both males and females.

**Fig. 2:**
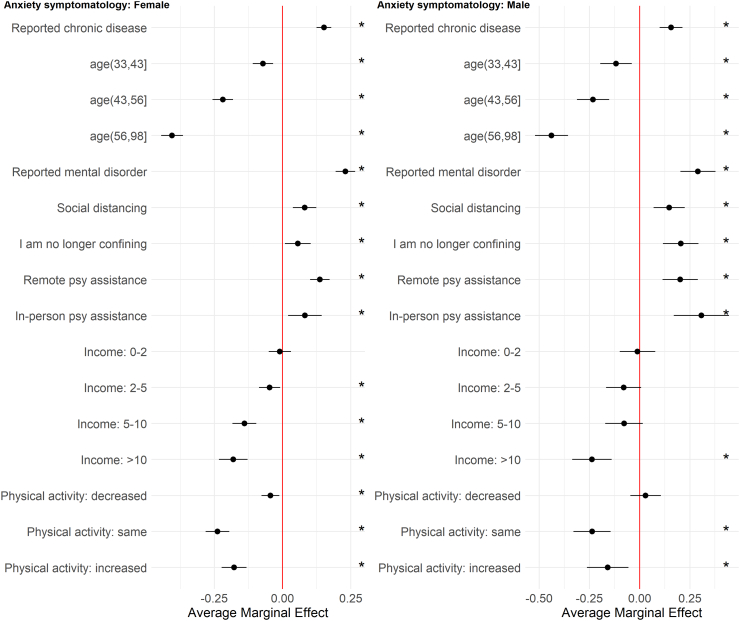
Results of glm models using Average Marginal Effect (AME). AME is the mean change in probability in the dependent variable for 1 unit increase in the explanatory variable. *, p < 0.05.

The presence of a chronic condition worsened anxiety during social isolation in both males and females. For females, there was a 16 % increase in anxiety symptoms for each unit increase in the value of the reported chronic disease variable (*p* < 0.05), and a 14 % increase in males (p < 0.05) (Fig. 2). Furthermore, younger subjects were more affected in a dose-response fashion, therefore the younger less likely to present with anxiety symptoms.

**Fig. 3:**
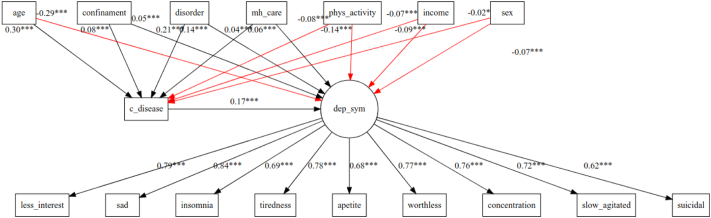
Structural equation model for depressive symptoms. Circles represent latent variables and boxes the measured variables. Straight arrows represent the direction of the relationship. Standardized estimated coefficients. Negative coefficients are in red, representing reduction of the value at the tip of the arrow. Residuals and variances were omitted. RMSEA = 0.044 (CI 0.043–0.046); SRMR = 0.045; TLI = 0.95. *, p < 0.05. (For interpretation of the references to color in this figure legend, the reader is referred to the web version of this article.)

**Fig. 4:**
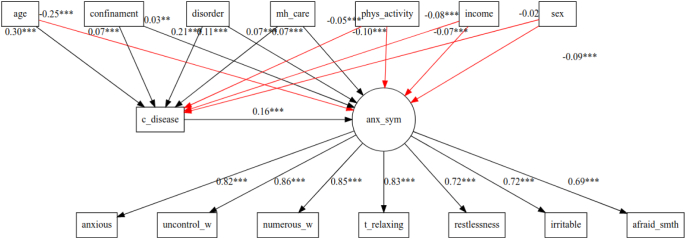
Structural equation model for anxiety symptoms. Circles represent latent variables and boxes the measured variables. Straight arrows represent the direction of the relationship. Standardized estimated coefficients. Negative coefficients are in red, representing reduction of the value at the tip of the arrow. Residuals and variances were omitted. RMSEA = 0.034 (CI 0.032–0.035); SRMR = 0.029; TLI = 0.87. *, p < 0.05. (For interpretation of the references to color in this figure legend, the reader is referred to the web version of this article.)

Subjects who at least kept their same physical activity level, regardless of whether men or women, had significantly less anxiety and depression symptoms during confinement. Those who performed some activity but reduced it during confinement tended still to have some protective effect, but at a level below those who kept their exercise schedule or increased it.

Finally, in-person contact with mental health services during the pandemic did not protect against anxiety and depression symptoms. For depression symptomatology in females the AME was 0.14 and 0.10 in males. For anxiety symptomatology the AME was 0.13 in females and in males 0.33.

### 3.1 Structural model for depressive symptoms

The existence of a chronic disease was significantly related to depressive symptoms during confinement on the COVID-19 pandemic. For each SD change in the variance of the variable encoding reported chronic condition there was a 0.17 SD rise in the variance of the variable encoding depressive symptoms. Variables included as covariates were significantly correlated with both the chronic disease and depression (*p* < 0.05) (Fig. 4). The strongest coefficient was between age and depressive symptoms, for each SD change in age there was a reduction of 0.29 SD in depressive symptoms. Therefore, the younger, the worse. Reported mental health assistance did not protect the subjects from presenting depressive symptomatology, whereas physical activity and income were protective. The model had an overall good fit, with robust versions of Mean Square Error of Approximation (RMSEA) = 0.044 (CI 0.043–0.046); Standardized Root Mean Square Residual (SRMR) = 0.045; Tucker-Lewis Index (TLI) = 0.95.

### 3.2 Structural model for anxiety symptoms

The existence of a chronic disease was significantly related to anxious symptomatology during social distance on the COVID-19 pandemic. The estimated coefficient from reported chronic disease to reported anxiety symptomatology was 0.16. Again, the included variables had a significant relationship with both the outcome and the presence of a chronic condition, except for sex which was not significant in relation to reported chronic disease. As with the depressive symptoms, increased age was protective against reported anxious symptoms (−0.25). Similar to what was found with depressive symptoms, reported mental health assistance did not reduce anxiety symptomatology reporting, whereas physical activity and income were protective. It is worth noting that physical activity had the strongest effect in reducing both anxiety and depressive symptoms. The model had an overall good fit, with robust versions of Mean Square Error of Approximation (RMSEA) = 0.034 (95%CI 0.032–0.035); Standardized Root Mean Square Residual (SRMR = 0.029); Tucker-Lewis Index (TLI) = 0.87. The findings from SEM confirmed what was found in the regression models.

## 4 Discussion

Using an on-line survey of over 14 thousand respondents in Brazil, we found that reported chronic disease was associated with increased reporting of both anxious and depressive symptoms during the pandemic. Women, younger and the poorer were most affected. All findings were confirmed in two structural equation models.

Having a chronic condition itself can increase worries related to health and therefore anxiety, but during a pandemic it can be especially intensified. It can be related to the impact on the health system, when resources for the care of different health problems are relocated to address the pandemic, but also due to the stress associated with the risk of being infected or the effect of the isolation (Kaparounaki et al., 2020; Wang et al., 2020). In addition, people with a previous chronic condition, such as diabetes and cardiovascular disease, are said to be at higher risk of severe disease or even death when infected by COVID-19. All these factors possibly explain the increased reporting of both anxious and depressive symptoms among our respondents who had a previous chronic disease.

Before the pandemic it was usually expected that older subjects tended to have more depressive symptoms (Maj et al., 2020). However, the COVID-19 pandemic increased unemployment, income instability, inflation and those factors normally impact the younger more than the older population. Younger Brazilians lost more jobs than older Brazilians according to the Brazilian Bureau of Statistics (IBGE, 2021). This might explain why we found an increased risk of both depression and anxiety symptoms among younger subjects confirming what was recently found in Austria (Pieh et al., 2020).

Brazil is a highly unequal country and the current pandemic has affected some ethnicities, minorities, and disadvantaged populations more than less vulnerable groups (Kapilashrami and Bhui, 2020). Our findings suggested a dose-response pattern, so that those with higher incomes showed lower depression and anxiety symptoms in both sexes, confirming a higher impact among those most vulnerable. This suggests that financial support is a possible avenue of action to improve the quality of life of the affected individuals, and to reduce psychological suffering. Previous studies, in Brazil, have shown an association of austerity measures with increased suicide rates (Alves et al., 2018; Machado et al., 2019) and decreased suicide associated with increased coverage of a cash transfer programme (Alves et al., 2018; Machado et al., 2019).

On the other hand, physical activity has been considered preventive and mitigatory for depression (Choi et al., 2020; Reichert et al., 2011) and anxiety (Yuenyongchaiwat, 2016). Subjects that at least maintained their regular fitness level during the pandemic presented with fewer symptoms of depression and anxiety. Staying active was fundamental to coping with the pandemic. This confirms the protective effect of physical activity and mental health during the pandemic found in Austria (Pieh et al., 2020), and suggests that stimulating physical activity in official health recommendations should help increase the population quality of life. Of course, we cannot exclude the alternate interpretation that mentally healthier people were more willing to keep physically active.

Some limitations are to be noted. The dataset was unbalanced in regard to sex, as women responded to the questionnaire in larger numbers. It was also unbalanced in regard to age and income. We stratified in order to partially control for these differences. There is a possibility of response bias and underestimation of chronic conditions, such that some individuals might be too ill to respond, leaving the study with the least severe cases. The level of missing information was relatively small for this dataset. This is a cross-sectional study, which cannot exclude issues with reverse causation and measurement bias in case more depressed respondents also reported more health problems.

This study is the first one that attempted to examine mental disorders, chronic clinical conditions and the pandemic in the Global South. The COVID-19 pandemic affected the population heterogeneously, individuals who already carried a previous chronic condition were at a higher risk of reporting anxiety and depression symptoms during the first wave. Additionally, we found that higher income was associated with fewer depressive and anxiety symptoms, highlighting how the COVID-19 pandemic can increase the vulnerability of the poorer population if measures are not put in place to protect them. Our findings can potentially inform practitioners and policy-makers in their recommendations to patients and to the public. The population with prevalent chronic conditions is on higher risk of depression and anxiety symptoms and therefore strategies for prevention of mental health disorders should be prioritized among the population with chronic comorbidities.

## Conflict of interest

Authors report no conflict of interest.

## Funding and role of the funding source

LFSCA, ESR, MLB, and DBM were funded by the 10.13039/501100000265Medical Research Council - UK, Grant no. MR/T03355X/1 during the study. LFSCA, FJOA, JAPA, MLB, ESR and DBM were also funded by the 10.13039/100000025National Institute of Mental Health, from the 10.13039/100000002NIH, Grant no. R01MH128911.

## CRediT authorship contribution statement

LFSCA led, designed, performed SEMs and wrote the paper. ESR led, performed analyses and wrote the paper. MPV participated in the discussion, adequacy and dissemination of the online questionnaire to recruit participants. CMPH acted in the recruitment of participants and in the critical review of the manuscript. JRB & DBM participated in the discussion, adapted and disseminated the online questionnaire to recruit participants and contributed to the writing of the manuscript. All authors reviewed the manuscript, approved the final version, and declared they had no competing interests.
